# PI3K/AKT Signaling in Breast Cancer Molecular Subtyping and Lymph Node Involvement

**DOI:** 10.1155/2019/7832376

**Published:** 2019-11-06

**Authors:** S. Bonin, D. Pracella, R. Barbazza, I. Dotti, S. Boffo, G. Stanta

**Affiliations:** ^1^Department of Medical Sciences, University of Trieste, Cattinara Hospital, Strada di Fiume 447, 34149 Trieste, Italy; ^2^Department of Gastroenterology, IDIBAPS, Hospital Clínic, CIBERehd, Barcelona, Spain; ^3^Sbarro Institute for Cancer Research and Molecular Medicine, Department of Biology, Temple University, 1900 N. 12th St., Philadelphia, PA 19122-6017, USA

## Abstract

Lymph node metastatic involvement persists to be among the most important predictors of recurrence and survival in breast carcinoma (BC). This study is aimed at investigating possible gene expression differences in primary BC between patients with or without lymph node involvement at the time of diagnosis. In a retrospective study, we investigated the potential prognostic role of 9 candidate biomarkers at the mRNA level in a cohort of 305 breast cancer patients, 151 lymph node-negative (LN-) and 154 lymph node-positive (LN+) individuals. The analyzed genes belonged to the RAS pathway (RAF1, ERBB2, PIK3CB, AKT1, AKT2, and AKT3), RB pathway (RB1 and CDK2), and cellular differentiation (KRT8). Their expression profiles were investigated by RT-qPCR and were correlated to immunohistochemically based molecular subtypes and BC clinical and pathological features. The differential expression of several genes in the primary tumor tissue was related to the LN involvement. Some of those genes, including PIK3CB, RB1, and AKT3, were more expressed in LN- BC patients, while some others, notably ERBB2 and AKT1, in LN+ ones. Among the candidate biomarkers, the expression levels of AKT isoforms influenced also patients' survival rates. In detail, higher expression levels of AKT1 and AKT2 negatively influenced overall patients' survival, and in particular, AKT2 expression levels defined a group of luminal B BC patients with shorter cancer-specific survival. On the contrary, longer cancer-specific survival was recorded in luminal A BC patients with higher expression levels of AKT3. That finding was also confirmed by Cox multivariate analysis. The same AKT3 resulted to be a possible candidate predictive biomarker for Tamoxifen response. In conclusion, our study highlighted the complex regulation of the PI3K/AKT pathway in BC and its differences in BC patients with and without lymph node involvement.

## 1. Background

Breast carcinoma (BC) encompasses a heterogeneous group of tumors with high variability at the molecular and morphological levels and clinical outcome [1, 2]. In the last few decades, advances have been made in BC detection and therapy through gene expression profiling, which has highlighted BC molecular complexity and has increased prognostication [3]. Subclassification of breast cancers into intrinsic subtypes has assisted in determining the need for adjuvant chemotherapy, particularly in patients with ER-positive diseases. In most hospitals worldwide, the clinical management of BC is mainly based on clinical-pathological features and the assessment of few surrogate biomarkers, namely, the estrogen receptor (ER), the progesterone receptor (PR), the human epidermal growth factor receptor-2 (ERBB2), and Ki67. The intrinsic BC subtypes have proven to be helpful for therapy decisions for node-negative ER+ breast cancer patients, especially with tumors grade 2 and stage 2.

Clinical variables, such as the nodal status and tumor size, remain highly clinically relevant even in the era of genomic testing and are likely to remain important also for evaluating the risk of relapse for patients with BC [4]. Although there is a strong association between molecular subtypes of BC and prognosis, a significant number of patients show similar features with distinct outcomes. It is estimated that 30–50% of patients with early to locally advanced breast cancer at diagnosis experience relapse, despite the use of adjuvant systemic treatment after surgery [5]. One of the major concerns regarding the utility of BC intrinsic subtypes is that BC prognostication is mainly addressed to young women at an early tumor stage, without lymph node involvement or with a maximum of 3 positive lymph nodes, so there is a lack of information for patients with a higher tumor stage.

In the present study, we have evaluated the mRNA expression profiles of 9 genes, 7 of which were tested in BC in other studies by our group [6, 7], in a cohort of young women aged 55 or less, both lymph node negative (LN-) and lymph node positive (LN+). The analyzed genes belong to signaling pathways that control major cellular processes, such as cell cycle regulation and the PI3K/AKT/TOR signaling pathway.

The genes analyzed in the present study are phosphatidylinositol 3 kinase (PIK3CB-p110*β*), v-AKT murine thymoma viral oncogene homolog 1 (AKT1), v-AKT murine thymoma viral oncogene homolog 2 (AKT2), v-AKT murine thymoma viral oncogene homolog 3 (AKT3), v-RAF-1 murine leukemia viral oncogene homolog 1 (RAF1), and keratin 8 (KRT8). The aim of this study is to investigate the possible impact of those genes in BC prognostication and characterization for lymph node involvement, the molecular subtyping, the clinical and pathological features, and a long follow-up period.

## 2. Methods

### 2.1. Patients

This is a population-based retrospective study. All patients resided in a province in the northeastern area of Italy. Inclusion criteria were (i) diagnosis of BC at least 15 years before the censoring date of the study (31^st^ of December 2008), (ii) invasive BC of stages I-III, (iii) age at diagnosis 55 years or younger, and (iv) availability of formalin-fixed and paraffin-embedded (FFPE) tissues. Cases with second primary breast cancer or other malignancies were excluded from the study. The patients enrolled and analyzed in the present study were characterized in a previous study through microarray analysis with immunohistochemical detection of ER, PR, Ki67, KRT8, KRT5/6, and vimentin [8].

The cohort included 305 patients; of these, 154 (50.4%) had lymph node involvement (LN+) at diagnosis. FFPE tissues of the primary tumor obtained by surgical treatment were used for molecular analysis. Clinical information was obtained from medical records. Tumors were reviewed, histologically and molecularly classified as already reported [8]. Classification of luminal A and B BC has been corrected based on the St. Gallen 2013 Consensus that recommended to use a cutoff of Ki67 < 20% for luminal A BC [9]. The patients' cohort was followed for a maximum of 25 years through the local cancer registry from diagnosis of BC to death or until the censoring date. This study was approved by the Ethical Committee of the University of Trieste as already described [8, 10]. Follow-up data were reported in previous studies [8, 10].

Briefly, patients were treated with mastectomy or breast-conserving surgery. All LN+ patients were treated with adjuvant chemotherapy regimens according to standard protocols as already reported [8]. ER-positive patients were submitted to hormone therapy with Tamoxifen. No specific treatment with trastuzumab was performed in HER2-positive patients because that therapy was not available at the time of diagnosis. Patients' clinical and pathological variables are reported in Table 1.

### 2.2. Gene Expression Profiling

The potential prognostic role of the 9 genes of interest was investigated by quantitative real-time PCR (qPCR). Briefly, total RNA was extracted from FFPE tissues after manual microdissection as previously described [11]. For each sample, 4 *μ*g of total RNA was treated with DNase as already described [12].

Complementary DNA (cDNA) synthesis was performed using 1.2 *μ*g of RNA and the Moloney murine leukemia virus (M-MLV) reverse transcriptase (Invitrogen, Karlsruhe, Germany) by random hexamer priming in a final volume of 20 *μ*l, as described in detail elsewhere [13].

Expression levels of RB1, CDK2, ERBB2, PIK3CB, AKT1, AKT2, AKT3, RAF1, and KRT8 were analyzed by qPCR using a Mastercycler® ep realplex (Eppendorf, Hamburg, Germany). PCR assays were performed in duplicate using TaqMan probes and the JumpStart™ Taq ReadyMix™ for quantitative PCR (Sigma-Aldrich, St. Louise, USA) according to the manufacturer's instructions. cDNA (30 ng for ACTB, CDK2, ERBB2, and AKT3; 40 ng for RAF1; 50 ng for AKT2; and 60 ng for RB1, PIK3CB, and AKT1) was amplified in a final volume of 20 *μ*l. Cycle conditions were (i) a denaturation step for 10 minutes at 95°C and (ii) 45 two-step cycles including 1 minute at 95°C for denaturation and 1 minute at 60°C for annealing-elongation. To exclude contamination, negative controls without cDNA were included in all analyses. RNA extracted from the breast cancer cell line MCF-7 was used as a positive control. Primer and probe sequences were created by the use of the Primer Express Software (Applied Biosystems, Darmstadt, Germany), and they are reported in Supplementary [Supplementary-material supplementary-material-1]. Gene expression levels were normalized using ACTB as the housekeeping gene, while RNA from 6 samples of the cohort were pooled and used as a calibrator. The relative quantification was determined using the method proposed by Livak and Schmittgen [14].

### 2.3. Statistical Analysis

The distribution of clinical-pathological features between LN-/LN+ and over the four molecular BC subtypes was investigated with the chi^2^ test. The ratio of the investigated genes at the mRNA level among lymph node involvement, the recurrence, and the molecular classification was analyzed with the Kruskal-Wallis test [15]. Spearman's rank correlation coefficient was measured to identify a possible correlation between protein and mRNA expression levels of ERBB2. To assess a trend across the ordered group, mRNA expression levels were submitted to an extension of the Wilcoxon rank-sum test, developed by Cuzick [16]. For survival analysis, the normalized ratios of the analyzed genes were dichotomized accordingly to their median value. In this way, patients with lower gene expression in comparison with the corresponding median were classified as having a lower status, while patients who presented higher values were classified as having the higher status of gene expression. The log-rank test was used to investigate whether each molecular and clinical-pathological variable affects the patients' survival. Afterward, the Cox proportional hazard regression method was applied for the analysis of pathological covariates (histologic type, stage, and grade of the tumor and age at diagnosis), molecular classification, and gene expression levels in the entire cohort of patients, to test the joint effects of the covariates on patients' survival. All statistical analyses were two sided, and values of *p* < 0.05 were considered statistically significant, although the Kruskal-Wallis test for more than two groups adjusted the significant value to a value lower than 0.05.

Logistic regression and ROC postestimation were used to predict lymph node involvement using the expression levels of specific genes in primary BC. All the statistical analyses were carried out with the package Stata/SE 12.0 (Stata, College Station, TX).

## 3. Results

### 3.1. Patients and Clinical-Pathological Tumor Characteristics

In this cohort of 305 patients, 151 resulted to be negative for axillary locoregional lymph node involvement (LN-) at the time of diagnosis and 154 showed positive axillary locoregional lymph nodes (LN+) as already reported [8, 10]. Patients' mean age at diagnosis was 47 years (range 26-55). No significant difference was detected for age at diagnosis between the groups defined by lymph node involvement [8]. The mean follow-up time was 14 years (range 0-25); in detail, it was 16 years (range 0-25) for the LN- group and 11 years (range 0-24) for the LN+ group. During the entire period of observation, 18 patients (6%) were lost at follow-up and 128 women (42%) died from breast carcinoma; of those, 39 (30%) were LN- and 89 (70%) were LN+. Recurrence was registered in 49 patients (33%) of the LN- group and 98 (69%) in the LN+ group. For 15 patients (5%) (4 LN- and 11 LN+), no information on the development of recurrences was found.

Molecular subtypes, as defined by immunohistochemistry (IHC) on the primary tumor, were as follows: 158 patients (52%) were luminal A, 84 women (28%) were luminal B, 38 were triple negative (TN) (12%), and 24 were HER2 positive (non-luminal) (8%) (Table 1). One case failed to be classified in any molecular subtypes as already reported [8].

Histological classification, tumor differentiation, and stage are reported in Table 1.

### 3.2. Relationship between Gene Expression and Clinical-Pathological Data

**Figure 1:**
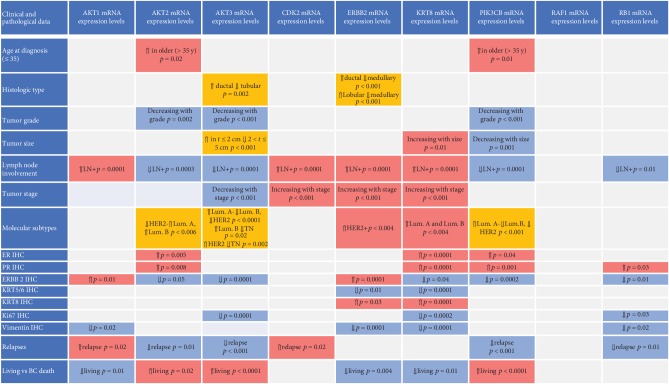
Graphical summary, representing associations among the mRNA expression of the analyzed genes and clinical-pathological variables. The red boxes indicate higher expression levels, the blue boxes indicate lower expression levels, and orange boxes are indicative of mixed associations. “Lum” stands for luminal and “IHC” for immunohistochemistry. IHC data are defined as positive or negative according to the already defined cutoffs.

The expression level of the mRNA of the 9 genes was investigated in relation to the following BC clinical-pathological factors: histological type (ductal, lobular, medullary, mucinous, and tubular), tumor grade (1, 2, and 3), tumor size (smaller than 2 cm, between 2 and 5 cm, and larger than 5 cm), lymph node involvement (yes or no), number of positive nodes (less or more than 3 lymph nodes) tumor stage (I, II, and III), presence of later recurrence (yes or no), age at diagnosis (younger or older than 35 years), and patient status at the end of follow-up (alive or dead from BC). A graphical summary of the results is reported in Figure 1. Detailed results are described in the supplementary file of the results.

### 3.3. Relationship between Gene Expression and Lymph Node Involvement

**Figure 2:**
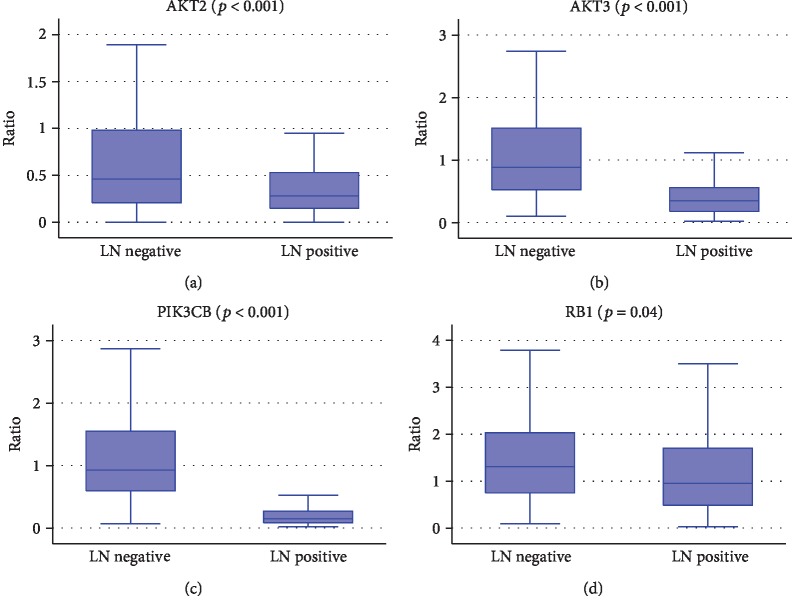
Box plot representing the mRNA expression levels (ratio of threshold cycles) of the genes that resulted to be significantly highly expressed in LN- BC in comparison to LN+ BC: (a) AKT2, (b) AKT3, (c) PIK3CB, and (d) RB1 (Kruskal-Wallis test).

**Figure 3:**
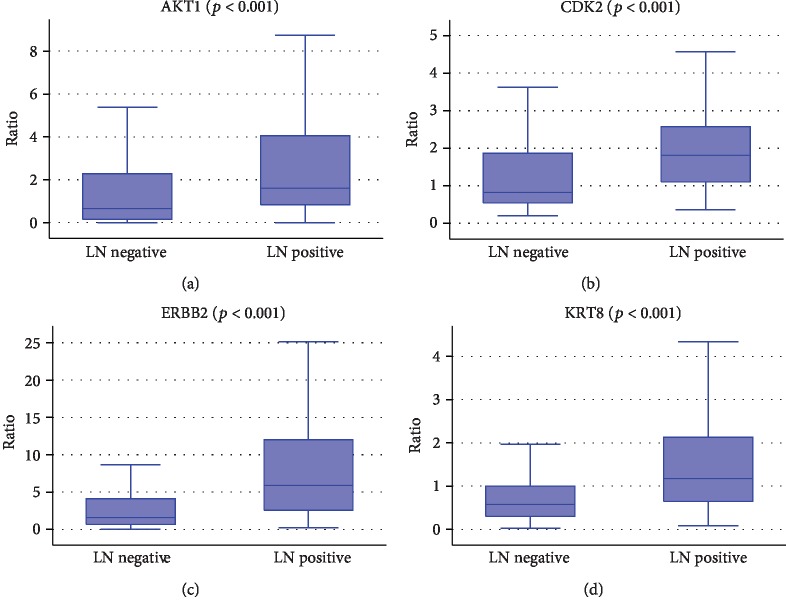
Box plot representing the mRNA expression levels (ratio of threshold cycles) of the genes that resulted to be significantly highly expressed in LN+ BC in comparison to LN- BC: (a) AKT1, (b) CDK2, (c) ERBB2, and (d) KRT8 (Kruskal-Wallis test).

Significant differences were found for eight out of nine candidate biomarkers according to lymph node involvement. In detail, the levels of expression of AKT2 (*p* < 0.001), AKT3 (*p* < 0.001), PIK3CB (*p* < 0.001), and RB1 (*p* = 0.01) were significantly higher in LN- patients as shown in Figure 2. Conversely, AKT1 (*p* < 0.001), CDK2 (*p* < 0.001), KRT8 (*p* < 0.001), and ERBB2 (*p* < 0.001) were more expressed in LN+ patients, irrespective of the number of involved lymph nodes (≤3 or >3) (Figure 3). Those results were confirmed also considering ER-positive tumors only. Logistic regression showed that AKT1 (*p* < 0.001), AKT3 (*p* = 0.009), ERBB2 (*p* = 0.002), PIK3CB (*p* < 0.001), and RB1 (*p* = 0.04) can describe lymph node involvement in our cohort (*p* < 0.001). A new variable was generated as a linear combination of the RT-qPCR ratio of the abovementioned genes as follows: LN involvement = 0.11∗ratioERBB2 − 9.79∗ratioPIK3CB − 1.69∗ratioAKT3 + 1.11∗ratioAKT1 − 0.69∗ratioRB1 to estimate the lymph node involvement. The cutoff was defined by ROC analysis at -1.9057. If the LN score was superior to that value, a lymph node involvement was predicted. The ratio represents for each gene included in the linear combination the value of the relative quantification for the specific gene as determined by RT real-time qPCR [14].

**Figure 4:**
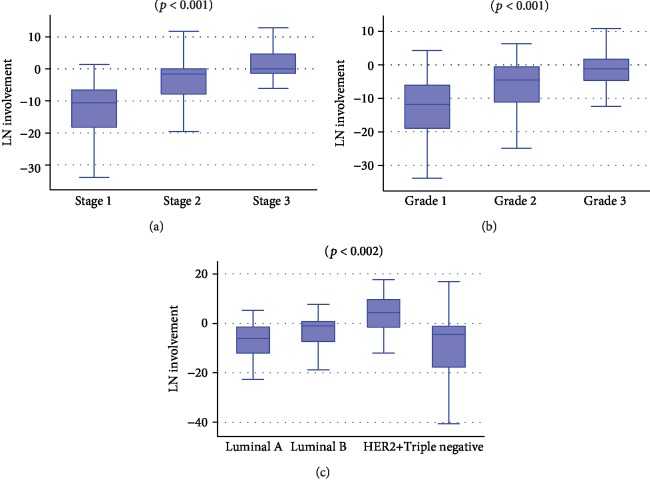
Box plot representing the distribution of the LN involvement variable as defined by the linear combination with respect of (a) BC stage, (b) BC grade, and (c) molecular subtypes. HER2+: HER2 positive (non-luminal). (Kruskal-Wallis and extended Wilcoxon rank-sum tests).

According to that calculation, we assessed BC-specific survival and the development of later recurrences. The LN involvement variable resulted to be significantly higher in patients who developed later recurrences (*p* < 0.0001) and discriminated among molecular subtypes (*p* < 0.0001) separating luminal A from luminal B (*p* < 0.0001), and luminal A from HER2 positive (non-luminal) (*p* < 0.0001) and HER2 positive (non-luminal) from TN BC (*p* < 0.0001). LN involvement increased from luminal A to HER2-positive patients (non-luminal) but was lower in TN patients. That variable also significantly increased with the tumor stage and grade (*p* < 0.0001 for both) as shown in Figure 4.

### 3.4. Patient Overall and Cancer-Specific Survival

**Figure 5:**
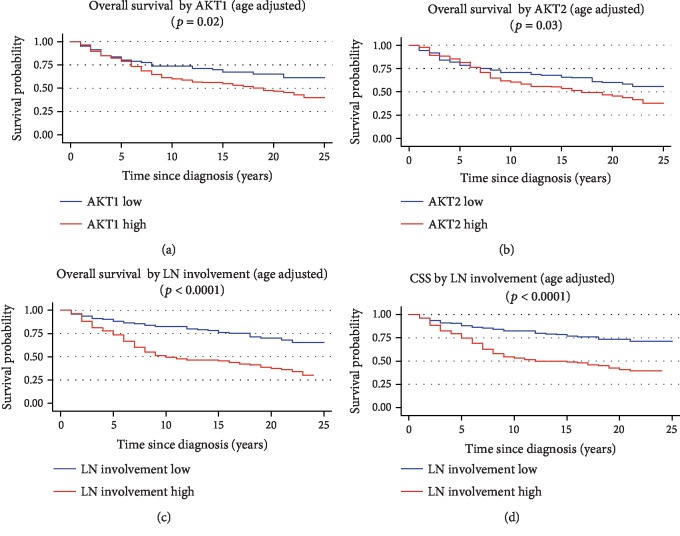
Age-adjusted Kaplan-Meyer overall survival curves for AKT1 expression (a), AKT2 expression (b), and LN involvement variable (c) in the entire cohort. Age-adjusted cancer-specific survival curves for LN involvement (d) in the entire cohort of patients. The gene expression was dichotomized in low expression and high expression for the median value of each transcript; the cutoff value for the LN involvement variable has been defined by ROC analysis. The graphs of the survivor function were adjusted to the mean age of patients (46.9 years). CSS: cancer-specific survival (*p* values on the graph refer to the log-rank test).

The expression levels of the candidate biomarkers were dichotomized according to their median value to investigate their influence on patients' survival. The LN involvement variable was dichotomized according to the cutoff obtained by ROC analysis. Over the entire cohort of BC patients, the overall survival seemed to be influenced by AKT1 (*p* = 0.02) and AKT2 expression levels (*p* = 0.03), as shown in Figures 5(a) and 5(b). Higher expression levels of those transcripts were associated with shorter overall survival. Nonetheless, neither AKT1 nor AKT2 expression confirmed to be independent variables that influence patients' survival as returned by the multivariate Cox regression analysis including clinical and pathological variables (age at diagnosis, stage, grade, and histological type) and AKT1 and AKT2 status as covariates. The LN involvement variable significantly influenced both overall survival and cancer-specific survival (CSS) of BC patients (Figures 5(c) and 5(d), respectively), as lower values of that variable were associated with longer survival.

**Figure 6:**
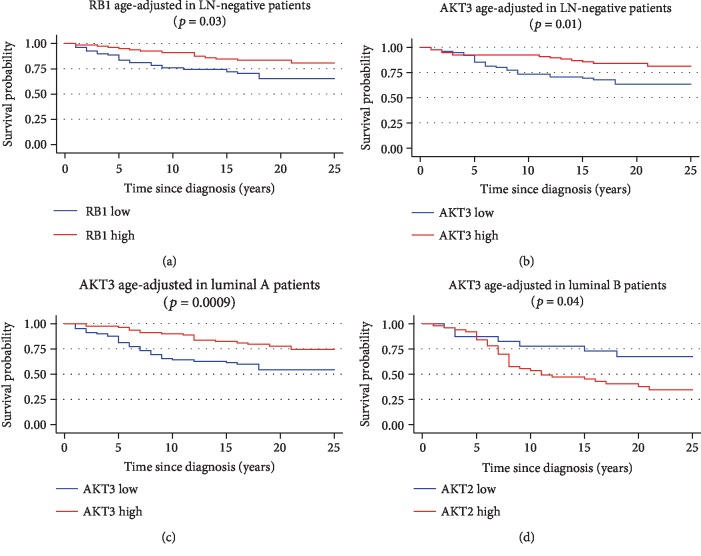
Age-adjusted Kaplan-Meyer cancer-specific survival curves for RB1 expression in LN- patients (a), AKT3 expression in LN- patients (b), AKT3 expression in luminal A patients (c), and AKT2 expression in luminal B patients (d). Graphs in (c) and (d) are irrespective of the LN+ and/or LN- group. The gene expression was dichotomized in low expression and high expression for the median value of each transcript. The graphs of the survivor function were adjusted to the mean age of patients (46.9 years). CSS: cancer-specific survival (*p* values on the graph refer to the log-rank test).

In LN- patients, RB1 and AKT3 seemed to have a protective effect on patients' cancer-specific survival (*p* = 0.03 and *p* = 0.01, respectively) as their higher expression levels were associated with longer survival (in univariate analysis) as shown in Figures 6(a) and 6(b). In multivariate Cox regression analysis, those two variables were not confirmed to influence independently CSS. In LN+ patients, none of the analyzed genes seemed to affect patients' survival.

**Figure 7:**
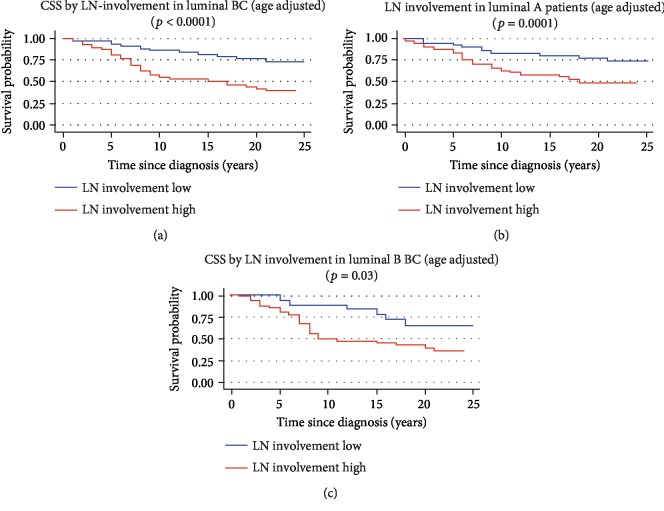
Age-adjusted Kaplan-Meyer cancer-specific survival curves for LN involvement variable in luminal BC patients (a), luminal A BC patients (b), and luminal B BC patients (c), irrespective of groups LN+ and/or LN-. LN involvement variable was dichotomized in low expression and high expression for its cutoff value which has been defined by ROC analysis. The graphs of the survivor function were adjusted to the mean age of patients (46.9 years). CSS: cancer-specific survival (*p* values on the graph refer to the log-rank test).

Patients' survival was investigated for the analyzed genes in the 4 molecular subtypes of BC, separately. A better cancer-specific survival was found for higher levels of AKT3 in luminal A BC patients (*p* = 0.0009, Figure 6(c)). Further investigation on the role of AKT3 in our cohort showed that estrogen- and progesterone-positive patients displayed longer cancer-specific survival where higher AKT3 levels (*p* = 0.006) were expressed supporting a possible predictive role for Tamoxifen response (see Supplementary file of results). Shorter cancer-specific survival was recorded in luminal B patients displaying higher levels of AKT2 (*p* = 0.03) (Figure 6(d)). The LN involvement variable was associated with CSS in luminal BC patients (*p* < 0.0001, Figure 7(a)), both in luminal A (*p* = 0.0001, Figure 7(b)) and in luminal B (*p* = 0.03, Figure 7(c)) BC patients. Longer survival for the high AKT3 status in luminal A patients was confirmed by Cox multivariate analysis including as covariates clinical and pathological variables (age at diagnosis, stage, grade, and histological type) and LN involvement variable, as reported in Table 2. The stage (*p* = 0.04) and grade (*p* = 0.05, borderline), age at diagnosis (*p* = 0.05, borderline), and AKT3 (*p* = 0.009) showed independent influence on patients' survival.

## 4. Discussion

This study is aimed at investigating the role of the mRNA expression levels of 9 genes belonging to signaling pathways that control major cellular processes in a cohort of 305 young patients (aged 55 years or less when diagnosed) affected by primary breast cancer for the molecular subtypes and clinical-pathological variables. The choice of genes was mostly related to the conclusions of our previous study [6] and lymph node involvement. Our results highlight a complex pattern of expression of the analyzed genes, which depends on lymph node involvement as well as on the tumor stage, grade, and molecular subtypes. The interplay of some analyzed genes in lymph node involvement is highlighted in our regression model, which points out a major contribution of AKT1 and also of ERBB2 in metastatic lymph node involvement as well as the protective effect of AKT3, PIK3CB, and RB1.

It is well known that LN involvement is a risk factor for metastasis in BC and that there is a higher risk of progression among patients with positive LN than expected in women with negative LN [17]. The expression levels of most genes analyzed in this study were differently represented in the group of patients with and without LN involvement, highlighting the difference of breast cancer for nodal involvement. Nonetheless, the expression levels of most genes were not independent variables affecting patients' survival, supporting a complex pattern in BC progression. Molecularly, LN involvement resulted to be related to the contribution of different genes at the primary tumor tissue level. Some of those genes were more expressed in LN- patients, such as PIK3CB, RB1, and AKT3, while some others in LN+ patients, such as ERBB2 and AKT1, as highlighted also in our regression model.

In detail, AKT1, KRT8, ERBB2, and CDK2 were highly expressed in the LN+ group, with no differences referred to the number of involved lymph nodes (>3 or ≤3). Those results were also valid considering only the luminal type of cancers. The LN+ group, in our cohort, had higher frequencies of HER2-positive (non-luminal) and luminal B tumors. Among the aforementioned genes, AKT1 was also highly expressed in recurrent BC patients and the same AKT1 with ERBB2 and KRT8 was also highly expressed in patients with breast cancer as a specific cause of death, likely because of the higher rate of HER2-positive (non-luminal) and luminal B types among women with that expression pattern. In agreement with us, both AKT1 and ERBB2 have already been found to be highly expressed in ER+ recurrent breast cancer [18] as possible genes involved in the mechanism of resistance to Tamoxifen. Data on CDK2 are in agreement with our previous study, which indicated that this cyclin-dependent kinase was significantly higher in patients with later recurrences [6]. Taking these observations into account, we could assume that higher mRNA expression levels of AKT1, CDK2, ERBB2, and KRT8 molecules could be an indicator of worse outcomes in our cohort of patients. We found, surprisingly and in disagreement with our previous work [6], that high KRT8 levels are indicative of poor outcome, despite the KRT8 role in cell differentiation and in characterization of luminal types of BC. KRT8 was highly expressed in LN+ BC and in patients dead from BC as it seemed to increase with the size and stage of the tumor. Overall, the adverse contribution of KRT8 in cancer is not novel and it seems to find a possible explanation in the phosphorylation of the protein. It has been shown, indeed, that phosphorylated KRT8 is required and is sufficient to induce keratin reorganization and consequently enhanced migration of human epithelial tumor cells [19]. In BC, in particular, higher KRT8 protein expression has been associated with lower survival probability as shown in the Protein Atlas website [20]. Also, Brotherick et al. detected higher KRT8 mRNA levels in node-positive patients as opposed to node-negative ones [21] which is in agreement with us. Additionally, KRT8 mRNA, but not K18 and MGL, was overexpressed in the blood of BC metastatic patients [22]. In our study, the higher expression levels of KRT8 can characterize luminal B BC, which are associated with a worse outcome among luminal tumors.

Higher expression of RB1, AKT3, and PIK3CB was detected in the LN- group and in patients without later recurrences. The results on RB1 agree with other studies that reported the loss of RB to be correlated with advanced disease and often with ER- subtypes of breast cancer [23]. Activation of the PI3K/AKT signaling pathway is observed in up to 81% of breast cancer patients [24]. In this study, we have analyzed the expression of the PI3K 110*β* subunit and the 3 isoforms of AKTs. The *β* isoform of the p110 catalytic subunit of PI3K seems to overlap the structure and the enzymatic function of the *α* isoform, but p110*α* and p110*β* isoforms have been reported to play distinct roles in cellular signaling, growth, and oncogenic transformation [25]. In our study, PI3K 110*β* expression was related to favorable prognostic factors, because it significantly decreased with the tumor size and grade; it was less expressed in LN-positive tumors, in relapsing BC, and it was highly expressed in luminal A BC and in living patients, supporting a protective role in BC progression. Our findings agree with the divergent roles of p110*α* and p110*β* isoforms in mammary gland tumorigenesis [25]. According to the hypothesis of Untermark and coauthors, a novel negative role of p110*β* in RTK signaling has been proposed that comprises a competition model in which the less active p110*β* competes with the more active p110*α* for receptor-binding sites. Thereby, downtuning the level of lipid kinase activity associated with receptors could explain the positive effect of p110*β* on our cohort.

The three highly homologous AKT isoforms (i.e., AKT1, AKT2, and AKT3) seem to play different or even functional opposing roles in our cohort of patients. AKT1 was related to shorter survival, and negative prognostic factors, mostly independently of the LN involvement as highlighted in Figure 1. Contrarily, AKT3 was associated with a good prognosis, while AKT2 seemed to be associated to luminal B BC. General activation of AKT has been shown to correlate with shorter disease-free survival [26] and to be associated with tumor progression [27]. However, emerging data indicate that the three highly homologous AKT isoforms may play different or even functional opposing roles in the regulation of migration and invasion [27, 28]. The role of the three isoforms in breast cancer has been reported with divergent results, because of the different methods used to detect them and of the samples analyzed. Recently, in breast cancer, AKT1 has been shown to be involved in the local tumor growth while AKT2 in the distant tumor dissemination [29]. In mouse and human breast cancer cells, the overactivation of AKT1 has been associated with ductal-like tumor growth [30] but other studies showed that AKT1 could inhibit epithelial-to-mesenchymal transition and BC metastasis [31–33]. Our results come out in favor of a negative role of AKT1 in BC. Regarding AKT2, our findings highlighted higher expression levels in ER-positive LN-negative tumors, but with a worse prognosis as shown by shorter survival in luminal B BC patients. In agreement with us, AKT2 has already been shown to modulate ER activity at multiple levels, with a key role in the regulation of ER function and its expression [34], and to promote cell migration and invasion [35]. Furthermore, in our cohort, higher levels of AKT2 were associated to lower tumor grade BC. This apparent discrepancy is because AKT2 is highly expressed in luminal tumors which in our cohort are the only ones including grade 1 BC. The functional role of AKT3 is the least examined which is therefore poorly understood. In our cohort of luminal BC patients, AKT3 resulted to have a protective role, even confirmed by the multivariate Cox regression. Our results highlight that ER- and PR-positive BC patients with higher AKT3 did not relapse during the follow-up and survived longer (see Supplementary file of results). Although it is not a direct proof, AKT3 could represent a possible predictive biomarker for Tamoxifen response, as all ER- and PR-positive patients of our cohort at the time of diagnosis were submitted to Tamoxifen treatment. No reports are supporting on that possible specific activity of AKT3 in ER- and PR-positive BC patients, but only a study on the interaction of AKT3 and estrogen function in MCF-7 cells where a constitutively active form of AKT3 was induced [36]. Thus far, AKT3 has been mostly studied in triple-negative breast cancers with conflicting results [24, 37–39]. Recently, Grottke and coworkers have highlighted the beneficial effect of AKT3 on BC cell lines. By knocking AKT3, but not AKT1 or AKT2, the authors promoted migration and the metastatic potential in the MDA-MB-231 cell lines [24]. Suyama et al. reported on a splice variant of AKT3, which promotes apoptosis and suppresses mammary tumorigenesis [38]. All those studies support our findings, although they are carried out in TN breast cancer cells. Our observation of the different contribution of AKTs in BC has been supported by gene expression profiling analysis (GEPIA -http://gepia.cancer-pku.cn) as shown in the supplementary file of results. In detail, AKT3 was significantly highly expressed in normal mammary glands if compared to BC tissue, an opposite trend resulted for AKT1, and no difference was noticed for AKT2.

In our cohort, cancer-specific survival seems to be influenced by a complex pattern by RB1 and AKT molecules, depending on the LN involvement and molecular subtypes (mostly luminal A and B). The reason could be the different relationship and dependence of AKT isoforms with specific effectors or activators as already shown for AKT2 and PTEN and ER [34, 35].

Even though the mutational rate of most genes analyzed in the present study is lower than 2.5% (see Supplementary file of results) if compared with 27% of PIK3CA, we acknowledge as a limitation in our study that the mutational status of PIK3CA has not been analyzed. Furthermore, no test set of samples has been analyzed to validate the possible use of the LN involvement variable in clinical practice and confirm our findings.

In conclusion, our findings highlight that breast cancers differed molecularly for LN involvement at the level of the primary tumor. By comparing genes' expression in these two groups of tumors, we found that AKT3 and RB1 influenced patients' survival in LN-negative patients. For molecular subtyping of BC, AKT3 resulted to be an independent favorable prognostic factor for luminal A BC patients and could represent a possible candidate biomarker to Tamoxifen response. Our results showed that high expression levels of AKT3 are associated with a better outcome and longer cancer-specific patients' survival rates in those patients who display the luminal A molecular class as well as in ER- and PR-positive ones. AKT2 and AKT3 mRNA expression levels can be useful in defining luminal BC with high and low risk of relapse, AKT2 defining worse prognosis and AKT3 longer survival. The possible interplay of the analyzed genes in lymph node involvement has been described in our regression model, which is also associated with BC recurrence and cancer-specific survival. Although it is not possible to discuss its possible clinical use, it shows that the PI3K/AKT pathway presents with the different and opposite contributions to lymph node involvement and disease progression.

## Figures and Tables

**Table 1:** Clinical-pathological characteristics of breast cancers involved in this study.

Factors	Total case study 305 (100%)	LN- 151 (49.5%)	LN+ 154 (50.5%)
Age (years)			
≤35	21 (6.9%)	4 (2.6%)	17 (11.0%)
>35	284 (93.1%)	147 (97.4%)	137 (89.0%)
Mean age (years) (range)	46.9 (26-55)	46.8 (32-55)	47.0 (26-55)
Histology			
Ductal	259 (84.9%)	118 (78.1%)	141 (91.6%)
Lobular	16 (5.2%)	9 (6.0%)	7 (4.5%)
Medullary	8 (2.6%)	8 (5.3)	—
Mucinous	4 (1.3%)	3 (2.0%)	1 (0.6%)
Tubular	7 (2.3%)	7 (4.6%)	—
Others	11 (3.6%)	6 (4.0%)	5 (3.2%)
Grade			
1	39 (12.8%)	33 (21.9%)	6 (3.9%)
2	144 (47.2%)	86 (57%)	58 (37.7%)
3	119 (39.0%)	31 (20.5%)	88 (57.1%)
Unknown	3 (1.0%)	1 (0.7%)	2 (1.3%)
Tumor size (cm)			
≤2	179 (58.7%)	107 (70.9%)	72 (46.8%)
2-5	100 (32.8%)	35 (23.2%)	65 (42.2%)
≥5	13 (4.3%)	1 (0.7%)	12 (7.8%)
Unknown	13 (4.3%)	8 (5.3%)	5 (3.2%)
Lymph nodes			
1-3 lymph nodes	90 (29.5%)	—	90 (58.4%)
≥4 lymph nodes	45 (14.8%)	—	45 (29.2%)
Unknown	19 (6.2%)		19 (12.3%)
Stage			
I	107 (35.1%)	107 (70.9%)	—
II	125 (41.0%)	42 (27.8%)	83 (53.9%)
III	72 (23.6%)	2 (1.3%)	70 (45.5%)
Unknown	1 (0.3%)	—	1 (0.6%)
Recurrence			
No	143 (46.9%)	98 (64.9%)	45 (29.2%)
Yes	147 (48.2%)	49 (32.5%)	98 (63.6%)
Unknown	15 (4.9%)	4 (2.6%)	11 (7.1%)
ER			
Negative	63 (20.7%)	25 (16.6%)	38 (24.7%)
Positive	242 (79.3%)	126 (83.4%)	116 (75.3%)
PR			
Negative	81 (26.6%)	27 (17.9%)	54 (35.1%)
Positive	223 (73.1%)	123 (81.5%)	100 (64.9%)
Unknown	1 (0.3%)	1 (0.7%)	—
ERBB-2			
Negative	242 (79.3%)	139 (92.0%)	103 (66.9%)
Positive	61 (20.0%)	11 (7.3%)	50 (32.5%)
Unknown	2 (0.7%)	1 (0.7%)	1(0.6%)
Ki67			
<20%	172 (56.4%)	108 (71.5%)	64 (41.6%)
≥20%	133 (43.6%)	43 (28.5%)	90 (58.4%)
KRT8			
Negative	28 (9.2%)	12 (7.9%)	16 (10.4%)
Positive	263 (86.2%)	134 (88.7%)	129 (83.8%)
Unknown	14 (4.6%)	5 (3.3%)	9 (5.8%)
KRT5/6			
Negative	230 (75.4%)	107 (70.9%)	123 (79.9%)
Positive	70 (23.0%)	42 (27.8%)	28 (18.2%)
Unknown	5 (1.6%)	2 (1.3%)	3 (1.9%)
Vimentin			
Negative	216 (70.8%)	97 (64.2%)	119 (77.3%)
Positive	60 (19.7%)	42 (27.8%)	18 (11.7%)
Unknown	29 (9.5%)	12 (7.9%)	17 (11.0%)

**Table 2:** Results of Cox multivariate analysis in luminal A patients.

Variables	Hazard ratio	95% CI	p
Histologic type	0.95	0.61-1.48	0.8
Stage	1.69	1.01-2.83	0.04
Grade	1.66	1.00-2.75	0.05
Age at diagnosis	1.06	1.00-1.13	0.05
AKT3	0.41	0.21-0.80	0.009
LN involvement variable	1.50	0.72-3.11	0.3

## Data Availability

The data that support the findings of this study are available on request from the corresponding author (SB).

## Abbreviations

HUGO: Gene Nomenclature Committee (https://www.genenames.org)
HER2: HER2 positive (non-luminal) breast cancer molecular subtype
TN: Triple negative.

## Supplementary Materials

Supplementary Materials Supplementary Materials include a file with details on primers and probe sequences used in the study and the description of results which are recalled in the manuscript. Supplementary table 1: primer and probe sequences. Supplementary figure 1: box plot of AKT2 (A), AKT3 (B), and PIK3CB (C) mRNA expression levels (ratio of threshold cycles) by tumor grade (Kruskal-Wallis test). Supplementary figure 2: box plot of AKT1 (A), AKT2 (B), and AKT3 (C) mRNA expression levels in breast cancer tissue (red box) vs normal mammary gland (grey box). “∗” indicates statistical significance (*p* < 0.05). Supplementary figure 3: box plot of AKT3 (B) mRNA expression levels (ratio of threshold cycles) by the development of later relapses during the follow-up period (Kruskal-Wallis test). Supplementary figure 4: age-adjusted Kaplan-Meyer survival curve of AKT3 expression in ER- and PR-positive patients, irrespective of lymph node involvement. Gene expression was dichotomized in low expression and high expression with respect of the median value of AKT3. The graphs of the survivor function were adjusted to the mean age of patients (46.9 years). CSS: cancer-specific survival (*p* values on the graph refer to log-rank test). Supplementary figure 5: representation of genomic alterations in breast cancer with respect of the genes involved in our regression analysis.
