# Supplementary material for: PI3K/AKT Signaling in Breast Cancer Molecular Subtyping and Lymph Node Involvement
Source: Dis Markers. 2019 Nov 6;2019:7832376. doi: 10.1155/2019/7832376 (PMC6875411; doi:10.1155/2019/7832376)
Supplement: Supplementary Materials — include a file with details on primers and probe sequences used in the study and the description of results which are recalled in the manuscript. Supplementary table 1: primer and probe sequences. Supplementary figure 1: box plot of AKT2 (A), AKT3 (B), and PIK3CB (C) mRNA expression levels (ratio of threshold cycles) by tumor grade (Kruskal-Wallis test). Supplementary figure 2: box plot of AKT1 (A), AKT2 (B), and AKT3 (C) mRNA expression levels in breast cancer tissue (red box) vs normal mammary gland (grey box). “∗” indicates statistical significance (p < 0.05). Supplementary figure 3: box plot of AKT3 (B) mRNA expression levels (ratio of threshold cycles) by the development of later relapses during the follow-up period (Kruskal-Wallis test). Supplementary figure 4: age-adjusted Kaplan-Meyer survival curve of AKT3 expression in ER- and PR-positive patients, irrespective of lymph node involvement. Gene expression was dichotomized in low expression and high expression with respect of the median value of AKT3. The graphs of the survivor function were adjusted to the mean age of patients (46.9 years). CSS: cancer-specific survival (p values on the graph refer to log-rank test). Supplementary figure 5: representation of genomic alterations in breast cancer with respect of the genes involved in our regression analysis. [file 7832376.f1.docx]

**MATERIAL AND METHODS**

Sequence of PCR primers and probes used for gene expression detection are listed in the Supplementary table 1.

Supplementary table 1: Primer and probe sequences

| **Gene** | **NCBI Reference Sequence** | **Sequences** | **Product size (bp)** |
| --- | --- | --- | --- |
| **ACTB** | NM_001101.3 | F: 5’-CGGCCCCTCCATCGT-3’  R: 5’-AAAGGGTGTAACGCAACTAAGTCAT-3’  MGBprobe: 5’-CACCGCAAATGCTTC-3’ | 66 |
| **RB1** | [NM_000321.2](http://www.ncbi.nlm.nih.gov/nucleotide/108773786?report=genbank&log$=nucltop&blast_rank=1&RID=39KFGR5B015) | F: 5’-TCCCATGTTGCTCAAAGAACCA-3’  R: 5’-CCGTGCACTCCTGTTCTGA-3’  MGBprobe: 5-TTCACCTCGAACACCC-3’ | 94 |
| **CDK2** | [NM_001798](http://www.ncbi.nlm.nih.gov/nucleotide/166362720?report=genbank&log$=nucltop&blast_rank=2&RID=39KX7ZH5014) | F: 5’-CCTCCCCTGGATGAAGATGGA-3’  R: 5’-CCGCTTGTTAGGGTCGTAGTG-3’  MGBprobe: 5’-CAGCATTTGCGATAACAA-3’ | 66 |
| **ERBB2** | NM_004448 | F: 5’-AGCGCTTTGTGGTCATCCA-3’  R: 5’-GCAGTGAGCGGTAGAAGGT-3’  MGBprobe: 5’-CCCAGCCAGTCCCTTG-3’ | 75 |
| **PIK3CB** | NM_006219 | F: 5’-TGCCGAGAGATTTTCCCACAAT-3’  R: 5’-CCTGAAGCTGAGCAACATCCT-3’  MGBprobe: ATTGACAGCAGTAATTTT | 85 |
| **AKT1** | NM_005163 | F: 5’-CCACTGTCATCGAACGCACCT-3’  R: 5’-CACAGTCTGGATGGCGGTTGT-3’  Probe: 5’-ATGTGGAGACTCCTGAGG-3’ | 77 |
| **AKT2** | NM_001626 | F: 5’-AAGCAGAGGCTTGGTGGG-3’  R: 5’-TTGATGCTGAGGAAGAACCTG-3’  Probe: 5’-CCAGCGATGCCAAGGAGG-3’ | 71 |
| **AKT3** | NM_005465.4 | F: 5’-TCGAGAGAGCGGGTGTTCT-3’  R: 5’-TGTAGATAGTCCAAGGCAGAGACAA-3’  MGBprobe R: 5’-ACGTGTGCGGTCCTC-3’ | 77 |
| **RAF1** | NM_002880.3 | F: 5’-TCAGGAATGAGGTGGCTGTTC-3’  R: 5’-TGTACCCCATGAAAAGCAGAA-3’  MGBprobe: 5’-CGCAAAACACGGCATG-3’ | 66 |
| **KRT8** | NM_002273.3 | F: 5’- GGCTCCAGGCTGAGATTGAG-3’  R: 5’- GCTCGGCATCTGCAATGG-3’  MGBprobe: 5’-CCAGAGGGCTTCCC-3’ | 72 |

**SUPPLEMENTARY RESULTS**

Hereafter only statistically significant results are reported referring to gene expression and demographical and pathological variables including histological type, tumor grade, tumor size and stage of the disease.

**Age at diagnosis** - When splitting the patients group with respect to age at diagnosis (if ≤35 or > 35 years old at the time of diagnosis), the expression levels of AKT2 and PIK3CB resulted to be significantly different between the two groups (p=0.02 and p=0.01, respectively) showing higher expression levels in patients aged more than 35 years at diagnosis.

**Histologic type**- AKT3 levels of expression differed significantly between ductal and tubular breast cancers, with higher expression levels in tubular BC (p= 0.002). ERBB2 transcripts were significantly less represented in medullary BC in comparison to ductal and lobular histotypes (p= 0.001).

**Tumor grade**-Tumor grade was significantly associated to the expression levels of AKT2 (p= 0.002), AKT3 (p< 0.001) and PIK3CB (p< 0.001). For all of those genes the expression levels decreased over the grade of the tumor with higher expression levels for grade 1 as shown in the supplementary figure 1.

**Supplementary figure 1**: Box plot of AKT2 (A), AKT3 (B) and PIK3CB (C) mRNA expression levels (ratio of threshold cycles) by tumor grade (Kruskal Wallis test).

**Tumor size**- KRT8 mRNA expression changed significantly with tumor size with a positive trend from smaller (≤2 cm) to larger tumors (≥ 5 cm) (p= 0.01). Conversely PIK3CB expression had a negative trend with tumor size, showing higher PIK3CB levels in smaller tumors (p= 0.001). AKT3 expression levels differed between smaller (≤2 cm) and tumors of intermediate measure (<2 and < 5 cm) with a higher level of expression for smaller ones (p< 0.001).

**Tumor stage**- The gene expression levels of CDK2, ERBB2 and KRT8 differed significantly with tumor stage showing a positive trend across the stage (p<0.001 for all). Contrarily AKT3 expression levels decreased across tumor stage (p< 0.001).

**Relationship between gene expression analysis and disease progression**

Gene expression levels were compared with variables describing the progression of the disease, among them the development of later recurrences and the information about the status of the patients at census (living or dead from BC). Following, only statistically significant results were listed.

**Later recurrences**- Higher expression levels of RB1 (p=0.01), AKT2 (p= 0.01), AKT3 (p<0.001) and PIK3CB (p<0.001) were seen in women without relapses during the follow-up. Contrarily, AKT1 and CDK2 expression levels were significantly higher in women who developed recurrences (p=0.02 for both).

**Disease progression**- Stratifying patients into living and dead from BC, higher levels of expression of PIK3CB (p<0.0001), AKT2 (p=0.02) and AKT3 (p<0.0001) were found in living women, while AKT1 (p=0.01), KRT8 (p=0.01) and ERBB2 (p=0.004) were higher in women who died of BC.

**Relationship between mRNA and protein expression by IHC**

The expression levels of the analyzed genes at the mRNA levels were compared with results obtained by IHC analysis. As previously reported [1] the proteins analyzed by IHC were: the surrogate biomarkers used in IHC for molecular subtype classification, namely ER, PR, Ki67 and ERBB2 and also Keratin 8, Keratin 5/6 and Vimentin. IHC results were dichotomized into negative and positive according to St. Gallen consensus (2013) or cutoff value already described [1].

AKT1 mRNA expression levels were lowered in both ERBB2 overexpressing and vimentin negative BC (p=0.01 and p=0.02 respectively). AKT2 mRNA was higher in ER and PR positive tumors (p=0.005 and p= 0.008 respectively), but lowered in HER2- positive (non-luminal) tumors (p= 0.05).

AKT3 mRNA expression levels were lowered in both Ki67 positive^[[1]](#footnote-1)^ and HER2- positive (non-luminal) BC (p=0.0001 for both).

CDK2 transcriptional levels did not vary between any group defined by positivity and negativity of IHC markers.

ERBB2 mRNA levels were highly expressed in HER2- positive (non-luminal) BC (p< 0.0001) as well as in KRT8 positive BC (p= 0.03), but it was lowered in BC positive for basal keratins and for vimentin (p= 0.01 and p<0.0001 respectively).

KRT8 mRNA expression levels were higher in ER and PR positive BC (p= 0.0001 for both) as well as in KRT8 positive BC (p= 0.0001), but it was lowered in HER2-positive (non-luminal) (p= 0.04) BC, in basal keratin positive (p=0.0001) and in vimentin positive BC (p=0.001).

PIK3CB mRNA was highly expressed in ER (p= 0.04) and PR (p= 0.001) positive BC and was lowered in HER2-positive (non-luminal) (p= 0.0002) and Ki67 positive (p< 0.0001) BC.

RAF1 mRNA expression levels did not vary between groups defined by positivity and negativity of IHC markers.

RB1 was highly expressed in progesterone positive BC (p=0.03), but it was less expressed in HER2 overexpressing BC (p=0.01) and in vimentin positive cancers (p=0.01).

By patients’ stratification into the 4 molecular subtypes, a significant difference in the expression levels of ERBB2, PIK3CB, AKT2, AKT3 and KRT8 was observed among groups. Higher ERBB2 mRNA levels were detected in HER2-positive (non-luminal) subtype (p<0.001), as expected. By mean of Spearman’s rank correlation, a positive correlation between ERBB2 protein detected in immunohistochemistry and ERBB2 mRNA level was found (ρ=0.51 and p<0.01). Higher expression levels of PIK3CB and AKT3 were found in luminal A class (p<0.001 for both) and KRT8 was mostly expressed in luminal B subtype (p<0.001). AKT2 transcripts were more represented in luminal tumors (both luminal A and B) if compared to HER2-positive (non-luminal) subtype (p=0.0002 and p=0.001 for luminal A and B, respectively).

**AKTs mRNA Expression levels in BC and Normal Mammary Tissue**

The expression profile of AKTs mRNA in breast cancer and normal mammary gland has been retrieved by GEPIA (<http://gepia.cancer-pku.cn)>. In case of AKT3 (Supplementary figure 2, panel (C)) a significant difference has been detected between normal and cancer tissues (p<0.05) with higher expression levels in normal mammary gland. That result supports our findings on AKT3.

**AKTs mRNA Expression in BC and Normal Mammary Tissue**


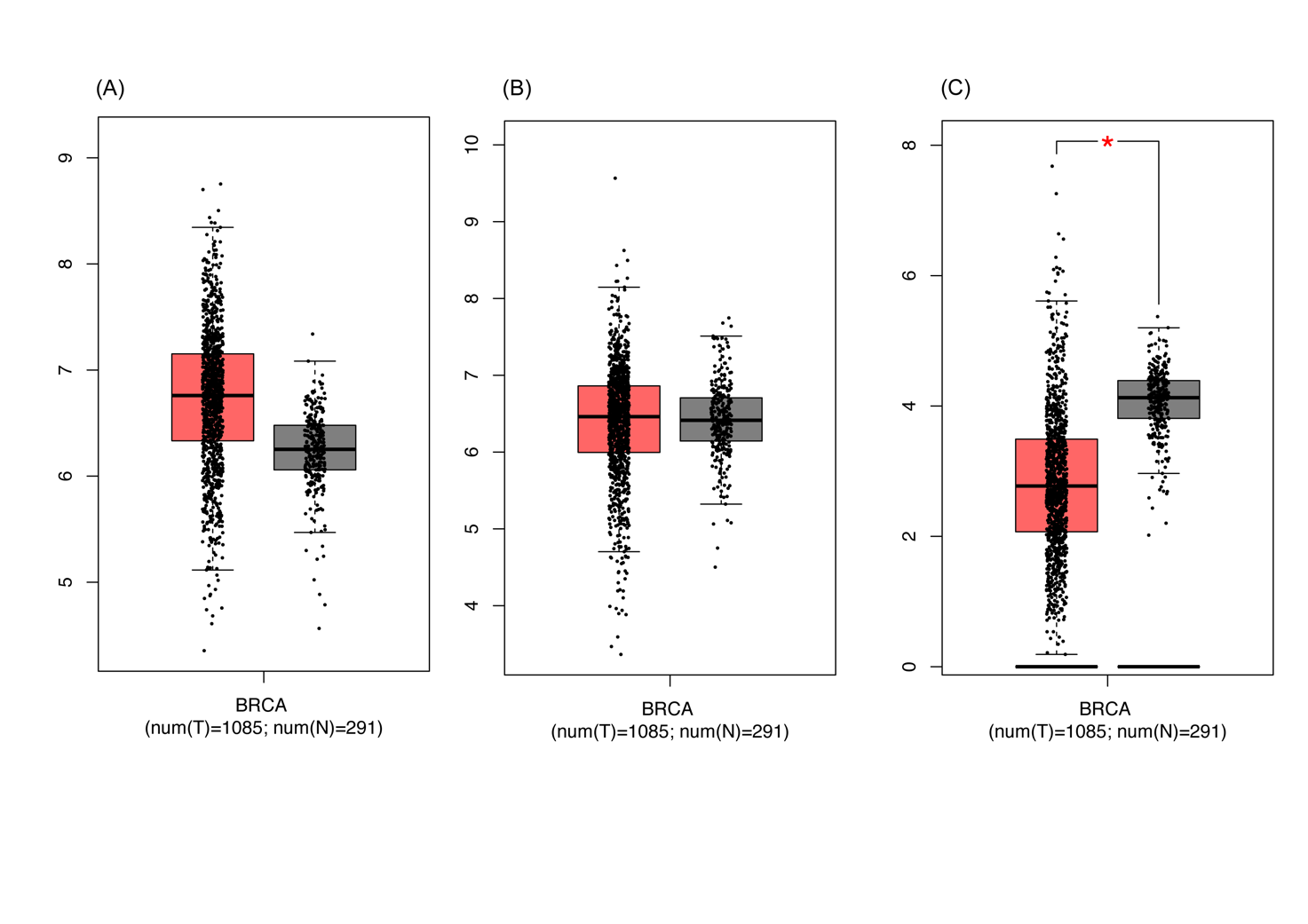


**Supplementary figure 2**: Box plot of AKT1 (A), AKT2 (B) and AKT3 (C) mRNA expression levels in breast cancer tissue (red box) vs normal mammary gland (grey box). “*” indicates statistical significance (p< 0.05)

**Associations of AKT3 expression levels in Estrogen and Progesterone positive patients**

To examine in depth the role of AKT3 in breast cancer patients we have investigated on its expression levels exclusively in ER and PR positive patients. Higher expression levels of AKT3 were detected in ER and PR positive patients who did not develop later recurrences during the follow-up as shown in supplementary figure 3, highlighting that AKT3 expression levels in ER and PR positive patients was linked to a better prognosis.

**Supplementary figure 3**: Box plot of AKT3 (B)) mRNA expression levels (ratio of threshold cycles) by the development of later relapses during the follow-up period (Kruskal- Wallis test).

To further confirm that hypothesis in the same cohort of patients, longer CSS survival was recorded for those patients expressing higher mRNA levels of AKT3 as shown in supplementary Fig 4. Taken together, it is likely that AKT3 expression could be related to Tamoxifen response as all ER and PR positive patients underwent Tamoxifen treatments and among them those without recurrences and longer cancer specific survival had higher AKT3 levels. However, we acknowledge that this is not actually a proof for the predictive role of AKT3 to Tamoxifen treatment.

**Supplementary figure 4:** Age-adjusted Kaplan-Meyer survival curve of AKT3 expression in ER and PR positive patients, irrespective of Lymph node involvement. Gene expression was dichotomized in low expression and high expression with respect of the median value of AKT3. The graphs of the survivor function were adjusted to the mean age of patients (46.9 years). CSS- Cancer Specific Survival, (p values on the graph refers to log-rank test).

**Mutational data on RB1, ERBB2, AKT1, AKT2, AKT3, PI3KCB from cbioportal.org**

Mutational rates of the analyzed genes involved in the logistic regression (ERBB2, PI3KCB, AKT1, AKT2, AKT3 and RB1) have been checked in cbioportal.org. Genomic alterations mostly account for gene amplification as shown by the red bar in the supplementary figure 3. The mutational rate for the analyzed gene was retrieved from 962 samples and it was: 2.5% for AKT1 (25 samples with AKT mutation), mostly the missense mutation E17K in PH domain; 0.4% for AKT2 (the 4 detected mutations are differently represented in the protein kinase domain); 0.5% for AKT3 (5 detected mutations: 3 missense and 2 truncating mutations, distributed in the PH domain (2) and in the protein kinase domain (3)); 2.5% for ERBB2 (ERBB2-26 samples mutated), 0.9% for PI3KCB (PIK3CB- 9 detected mutations distributed along the gene) and 2.2% for RB1 (25 samples with RB mutations widely distributed along the gene).


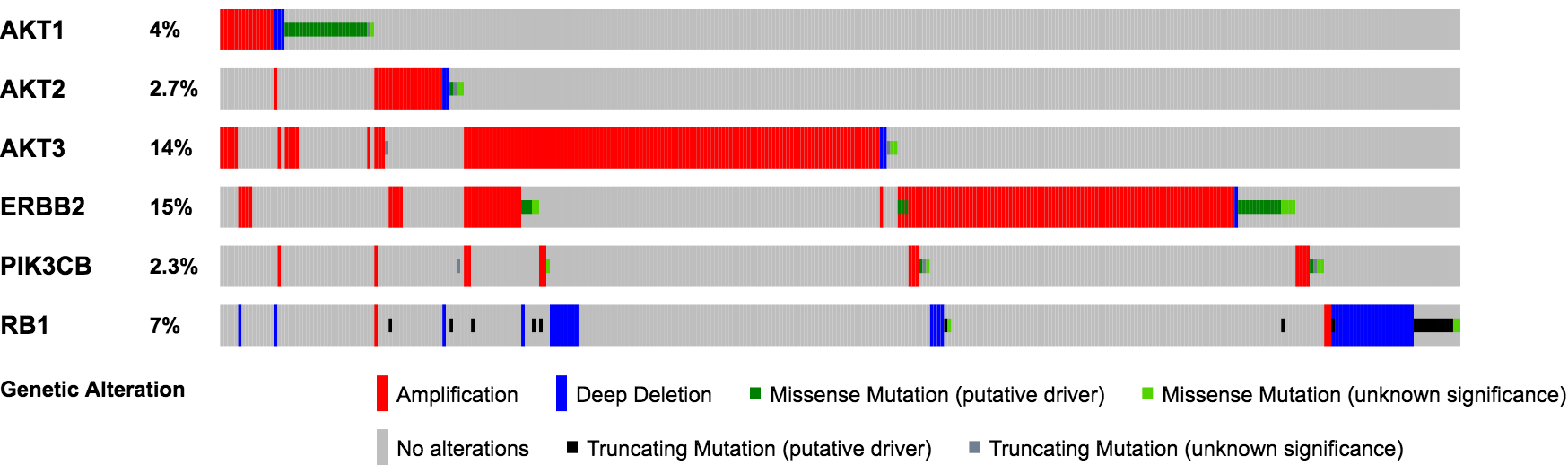


**Supplementary figure 5**: Representation of genomic alterations in Breast Cancer with respect of the genes involved in our regression analysis ([http://www.cbioportal.org)](http://gepia.cancer-pku.cn)).

**References**

1. Pracella D, Bonin S, Barbazza R, Sapino A, Castellano I, Sulfaro S, Stanta G: **Are breast cancer molecular classes predictive of survival in patients with long follow-up?** *Disease markers* 2013, **35**(6):595-605.

1. Ki67 cut-off is 20% as defined by the St Gallen 2013 consensus. [↑](#footnote-ref-1)
